# METTL3-mediated m^6^A modification of HMGA2 mRNA promotes subretinal fibrosis and epithelial–mesenchymal transition

**DOI:** 10.1093/jmcb/mjad005

**Published:** 2023-03-21

**Authors:** Yuwei Wang, Yuhong Chen, Jian Liang, Mei Jiang, Ting Zhang, Xiaoling Wan, Jiahui Wu, Xiaomeng Li, Jieqiong Chen, Junran Sun, Yifan Hu, Peirong Huang, Jingyang Feng, Te Liu, Xiaodong Sun

**Affiliations:** Department of Ophthalmology, Shanghai General Hospital, Shanghai Jiao Tong University School of Medicine, Shanghai 200080, China; Shanghai Key Laboratory of Ocular Fundus Diseases, Shanghai 200080, China; Department of Ophthalmology, Shanghai General Hospital, Shanghai Jiao Tong University School of Medicine, Shanghai 200080, China; Shanghai Key Laboratory of Ocular Fundus Diseases, Shanghai 200080, China; Shanghai Key Laboratory of Ocular Fundus Diseases, Shanghai 200080, China; Department of Ophthalmology, Shanghai General Hospital, Shanghai Jiao Tong University School of Medicine, Shanghai 200080, China; Shanghai Key Laboratory of Ocular Fundus Diseases, Shanghai 200080, China; Department of Ophthalmology, Shanghai General Hospital, Shanghai Jiao Tong University School of Medicine, Shanghai 200080, China; Shanghai Key Laboratory of Ocular Fundus Diseases, Shanghai 200080, China; Department of Ophthalmology, Shanghai General Hospital, Shanghai Jiao Tong University School of Medicine, Shanghai 200080, China; Shanghai Key Laboratory of Ocular Fundus Diseases, Shanghai 200080, China; Department of Ophthalmology, Shanghai General Hospital, Shanghai Jiao Tong University School of Medicine, Shanghai 200080, China; Shanghai Key Laboratory of Ocular Fundus Diseases, Shanghai 200080, China; Department of Ophthalmology, Shanghai General Hospital, Shanghai Jiao Tong University School of Medicine, Shanghai 200080, China; Shanghai Key Laboratory of Ocular Fundus Diseases, Shanghai 200080, China; Department of Ophthalmology, Shanghai General Hospital, Shanghai Jiao Tong University School of Medicine, Shanghai 200080, China; Shanghai Key Laboratory of Ocular Fundus Diseases, Shanghai 200080, China; Department of Ophthalmology, Shanghai General Hospital, Shanghai Jiao Tong University School of Medicine, Shanghai 200080, China; Shanghai Key Laboratory of Ocular Fundus Diseases, Shanghai 200080, China; Department of Ophthalmology, Shanghai General Hospital, Shanghai Jiao Tong University School of Medicine, Shanghai 200080, China; Shanghai Key Laboratory of Ocular Fundus Diseases, Shanghai 200080, China; Department of Ophthalmology, Shanghai General Hospital, Shanghai Jiao Tong University School of Medicine, Shanghai 200080, China; Shanghai Key Laboratory of Ocular Fundus Diseases, Shanghai 200080, China; Department of Ophthalmology, Shanghai General Hospital, Shanghai Jiao Tong University School of Medicine, Shanghai 200080, China; Shanghai Key Laboratory of Ocular Fundus Diseases, Shanghai 200080, China; Shanghai Geriatric Institute of Chinese Medicine, Shanghai University of Traditional Chinese Medicine, Shanghai 200031, China; Department of Ophthalmology, Shanghai General Hospital, Shanghai Jiao Tong University School of Medicine, Shanghai 200080, China; Shanghai Key Laboratory of Ocular Fundus Diseases, Shanghai 200080, China; National Clinical Research Center for Eye Diseases, Shanghai 200080, China; Shanghai Engineering Center for Visual Science and Photomedicine, Shanghai 200080, China

**Keywords:** METTL3, *N* ^6^-methyladenosine, epithelial–mesenchymal transition, subretinal fibrosis, HMGA2

## Abstract

Subretinal fibrosis is a major cause of the poor visual prognosis for patients with neovascular age-related macular degeneration (nAMD). Myofibroblasts originated from retinal pigment epithelial (RPE) cells through epithelial–mesenchymal transition (EMT) contribute to the fibrosis formation. *N*^6^-Methyladenosine (m^6^A) modification has been implicated in the EMT process and multiple fibrotic diseases. The role of m^6^A modification in EMT-related subretinal fibrosis has not yet been elucidated. In this study, we found that during subretinal fibrosis in the mouse model of laser-induced choroidal neovascularization, METTL3 was upregulated in RPE cells. Through m^6^A epitranscriptomic microarray and further verification, high-mobility group AT-hook 2 (HMGA2) was identified as the key downstream target of METTL3, subsequently activating potent EMT-inducing transcription factor SNAIL. Finally, by subretinal injections of adeno-associated virus vectors, we confirmed that METTL3 deficiency in RPE cells could efficiently attenuate subretinal fibrosis *in vivo*. In conclusion, our present research identified an epigenetic mechanism of METTL3–m^6^A–HMGA2 in subretinal fibrosis and EMT of RPE cells, providing a novel therapeutic target for subretinal fibrosis secondary to nAMD.

## Introduction

Age-related macular degeneration (AMD) is one of the leading causes of blindness in the elderly (Wong et al., 2014). Approximately 90% of AMD-related vision loss results from neovascular AMD (nAMD), which is characterized by choroidal neovascularization (CNV) (Lim et al., 2012). Subretinal fibrosis is the end stage of the natural history of nAMD. The scar leads to permanent photoreceptor damage and irreversible visual impairment (Gillies et al., 2020). Even with the injection of anti-vascular endothelial growth factor, which is the first-line therapy for nAMD, 20.37%–61.4% of patients still respond poorly and progress into subretinal fibrosis (Bloch et al., 2013; Rofagha et al., 2013; Miere et al., 2015; De Rosa et al., 2020; Gillies et al., 2020; Teo et al., 2020). Platelet-derived growth factor (PDGF) has been regarded as a potential target to attenuate subretinal fibrosis (Liu et al., 2020b). However, the phase-3 study (NCT01940900) failed to confirm the efficacy of the anti-PDGF agent recently, implying the importance and urgency of seeking other therapies based on the mechanism of fibrosis formation (Dunn et al., 2017; Rosenfeld and Feuer, 2018).

The pathogenic mechanisms of subretinal fibrosis secondary to nAMD are complicated. Myofibroblasts (active fibroblasts) are key cells in fibrosis development, transdifferentiated from a variety of cell types (Ishikawa et al., 2016a; Little et al., 2018). As one of the sources, retinal pigment epithelial (RPE) cells proliferating and undergoing epithelial–mesenchymal transition (EMT) have increasingly been shown as a critical contributor to subretinal fibrosis (Ishikawa et al., 2016a; Little et al., 2018). During the EMT process, RPE cells lose the junctions and polarity and transdifferentiate into motile mesenchymal cells, giving rise to the accumulation and remodeling of extracellular matrix (ECM) components (Grisanti and Guidry, 1995). Specifically, EMT is a process triggered by several inducers and regulated by a variety of transcription factors (TFs) and signaling pathways, which form a complex and unclarified regulatory network (Shu et al., 2020; Zhou et al., 2020a). Numerous efforts have been made to explore the pivotal role of EMT-inducing transcription factors (EMT-TFs) in driving the expression of EMT-regulatory genes (Hirasawa et al., 2011; Palma-Nicolas and Lopez-Colome, 2013; Li et al., 2014a). However, transcriptional regulation cannot fully explain the reversible cellular plasticity during EMT.

There is a growing interest in the research of the roles of epigenetic reprogramming in EMT, as it provides a more stable, long-term, and reversible regulation, which is critical in the context of cellular plasticity involved in EMT (Skrypek et al., 2017). Currently, the understanding of epigenetic regulation of EMT-related factors in RPE cells is very limited. *N*^6^-Methyladenosine (m^6^A) is the most abundant epigenetic modification in eukaryotic messenger RNAs (mRNAs), affecting gene expression by regulating mRNA splicing, export, stability, and translation. m^6^A modification is dynamic and reversible, installed by RNA methyltransferase complex (also called m^6^A ‘writers’), which is composed of methyltransferase-like 3 (METTL3), METTL14, and Wilms tumor 1-associated protein (Zeng et al., 2020; Zhou et al., 2020b). METTL3 is the only catalytic subunit of the complex and has been recently reported to regulate EMT in the progression and invasion of human cancers through triggering translation of SNAIL mRNA (Lin et al., 2019; Yue et al., 2019; Luo et al., 2020). Besides, m^6^A ‘eraser’ ALKBH5 negatively regulated SNAIL in EMT-driven renal fibrosis (Ning et al., 2020). Recently, Ma et al. (2021) suggested that METTL3 overexpression inhibits EMT of ARPE-19 cells *in vitro* and suppresses the proliferative vitreoretinopathy (PVR) process *in vivo*. However, the role of RNA m^6^A methylation in EMT-mediated subretinal fibrosis has not yet been elucidated as far as we know.

High-mobility group AT-hook 2 (HMGA2) is a non-histone architectural TF that positively regulates the EMT process in a series of fibrotic diseases (type 2 EMT) and cancer metastasis (type 3 EMT) (Liang et al., 2014; Hou et al., 2018; Zhu et al., 2019; Song et al., 2021; Wang et al., 2021). HMGA2 binds to AT-rich DNA sequences with its three AT hooks to alter chromatin architecture, thereby affecting the expression of EMT-related proteins in different ways (Thuault et al., 2008; Tan et al., 2012, 2015; Li et al., 2014b, 2017). For instance, HMGA2 not only transcriptionally activates EMT-TFs, such as SNAIL, SLUG, and TWIST (Thuault et al., 2008; Tan et al., 2012; Li et al., 2014b, 2017), but also epigenetically silences E-cadherin, a prototypical epithelial cell marker of EMT (Tan et al., 2015). The drivers of HMGA2 upregulation have also been investigated extensively, including indirect regulation of METTL3 (Rong et al., 2021; Zhao et al., 2021). Recent studies reported that HMGA2 was upregulated in RPE cells undergoing EMT induced by transforming growth factor-beta 2 (TGF-β2) (Sripathi et al., 2019, 2021). However, the role of HMGA2 in the regulatory network and the underlying mechanism remain to be clarified.

In the present study, we demonstrated that upregulated METTL3 was essential for promoting subretinal fibrosis and EMT of mouse primary RPE cells. HMGA2 was identified as the critical target of METTL3 in RPE cells undergoing EMT. The METTL3–m^6^A–HMGA2 axis subsequently enhanced another critical EMT-TF SNAIL. The results revealed the regulation of METTL3-mediated m^6^A methylation in EMT-mediated subretinal fibrosis, implying its potential as a novel target for subretinal fibrosis secondary to nAMD.

## Results

### METTL3 is upregulated in EMT-mediated subretinal fibrosis after laser photocoagulation

**Figure 1:**
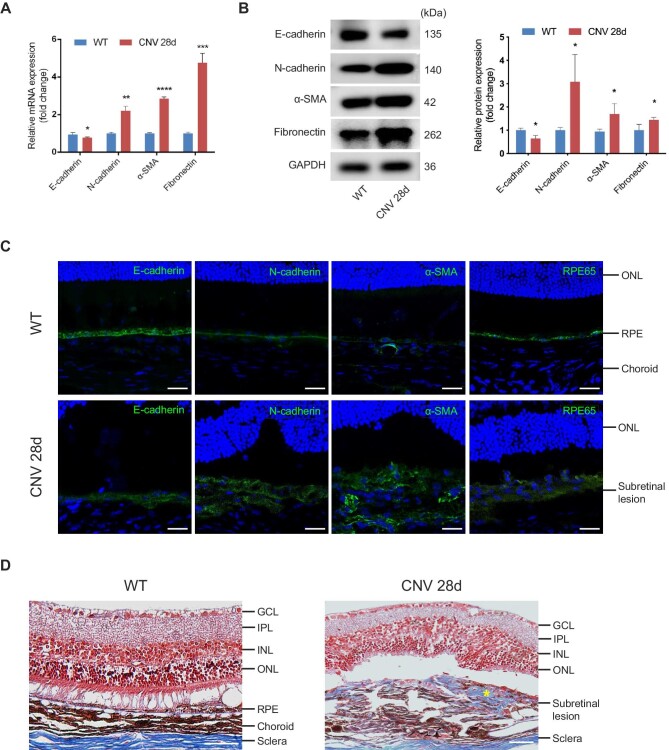
EMT of RPE cells and subretinal fibrosis are observed on Day 28 after laser photocoagulation. (**A** and **B**) The mRNA (**A**) and protein (**B**) levels of EMT-related markers, including E-cadherin, N-cadherin, α-SMA, and Fibronectin, in RPE–choroid complexes were detected on Day 28 after laser injury. (**C**) Immunofluorescence analysis showed the expression of EMT-related proteins (E-cadherin, N-cadherin, and α-SMA) and RPE-specific marker (RPE65). Scale bar, 25 µm. (**D**) Masson's trichrome staining revealed subretinal deposition of collagen content that stained blue (yellow star) in the CNV group. Scale bar, 50 µm. Data present mean ± SD of three independent experiments. Student's *t*-test, *P < 0.05, **P < 0.01, ***P < 0.001, ****P < 0.0001. GCL, ganglion cell layer; IPL, inner plexiform layer; INL, inner nuclear layer; ONL, outer nuclear layer.

We established a laser-induced CNV model to mimic the natural pathogenic course involved in patients with subretinal fibrosis secondary to nAMD. We examined the subretinal fibrosis and EMT on Day 28 after laser injury according to our previous study (Luo et al., 2018). Markers including E-cadherin, N-cadherin, α-SMA, and Fibronectin were evaluated at mRNA and protein levels. As shown in Figure 1A and B, mesenchymal markers (N-cadherin, α-SMA) and ECM protein (Fibronectin) were upregulated, while epithelial marker (E-cadherin) was downregulated after the laser. Consistently, immunofluorescence staining showed that RPE cells lost epithelial phenotype and RPE characteristics, transitioning into a mesenchymal phenotype along with subretinal fibrosis development (Figure 1C). Besides, cells with double staining of RPE65 and α-SMA were detected in the subretinal region, indicating the EMT process of RPE cells in this model (Supplementary Figure S1). Masson's trichrome staining demonstrated a marked subretinal deposition of collagen in the CNV group compared with the wild-type (WT) group (Figure 1D).

**Figure 2:**
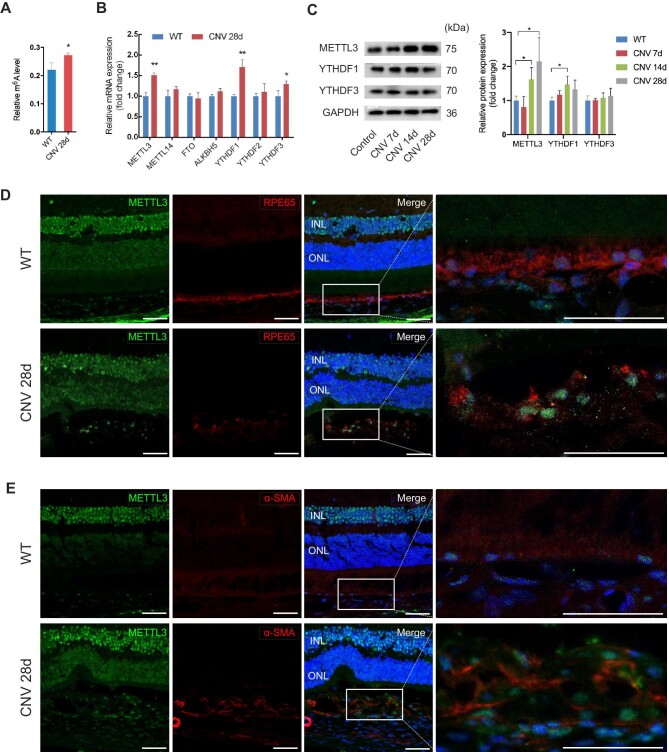
RNA m^6^A methylation and METTL3 expression are upregulated in EMT of RPE cells and fibrotic scar formation. (**A**) Total m^6^A levels of RPE–choroid complexes from the CNV group and control group were detected on Day 28 after laser injury. (**B**) The mRNA levels of several m^6^A enzymes were measured by qRT-PCR, including ‘writer’ (METTL3 and METTL14), ‘eraser’ (FTO and ALKBH5), and ‘reader’ (YTHDF1, YTHDF2, and YTHDF3). (**C**) The protein levels of METTL3, YTHDF1, and YTHDF3 were quantified by western blotting on Days 7, 14, 21, and 28 after laser photocoagulation. (**D** and **E**) Triple immunofluorescence staining for RPE65 (**D**), α-SMA (**E**), and METTL3 revealed a small amount of METTL3^+^RPE65^+^ cells in the subretinal region. Scale bar, 25 µm. Data present mean ± SD of three independent experiments. Student's *t*-test, *P < 0.05, **P < 0.01.

To study the role of RNA m^6^A in subretinal fibrosis, total m^6^A levels were measured by m^6^A RNA Methylation Quantification Kit. Compared with that in normal tissues, the m^6^A level of total RNA in RPE–choroid complexes was slightly more abundant on Day 28 after the laser (Figure 2A). The quantitative real-time polymerase chain reaction (qRT-PCR) was performed to detect the expression of several m^6^A enzymes, including ‘writer’ (METTL3 and METTL14), ‘eraser’ (FTO and ALKBH5), and ‘reader’ (YTHDF1, YTHDF2, and YTHDF3), among which METTL3, YTHDF1, and YTHDF3 were upregulated on Day 28 of laser-induced CNV (Figure 2B). To further confirm our observations, we measured the protein level at different points in time after the laser. The protein expression of METTL3 was not changed on Day 7, whereas it was enhanced on Days 14 and 28 (Figure 2C). A slight increase in YTHDF1 protein was shown on Day 14, while YTHDF3 protein remained unchanged during fibrotic scar formation (Figure 2C). Therefore, we focused on the role of METTL3 in subretinal fibrosis in this study. To figure out in which cell type METTL3 was increased, we performed immunofluorescence co-staining of METTL3 and several cell markers including RPE65, α-SMA, isolectin B4 (IB4), and F4/80 (Figure 2D and E; Supplementary Figure S2). We found that increased METTL3 was located in the RPE65-positive and α-SMA-positive cells (Figure 2D and E). Despite downregulation of RPE65 after laser injury due to the dedifferentiation of RPE cells, we observed a small amount of METTL3^+^RPE65^+^ cells. The data suggested that METTL3 was involved in EMT of RPE cells and fibrotic scar formation.

### METTL3 is involved in primary mouse RPE cells undergoing EMT

**Figure 3:**
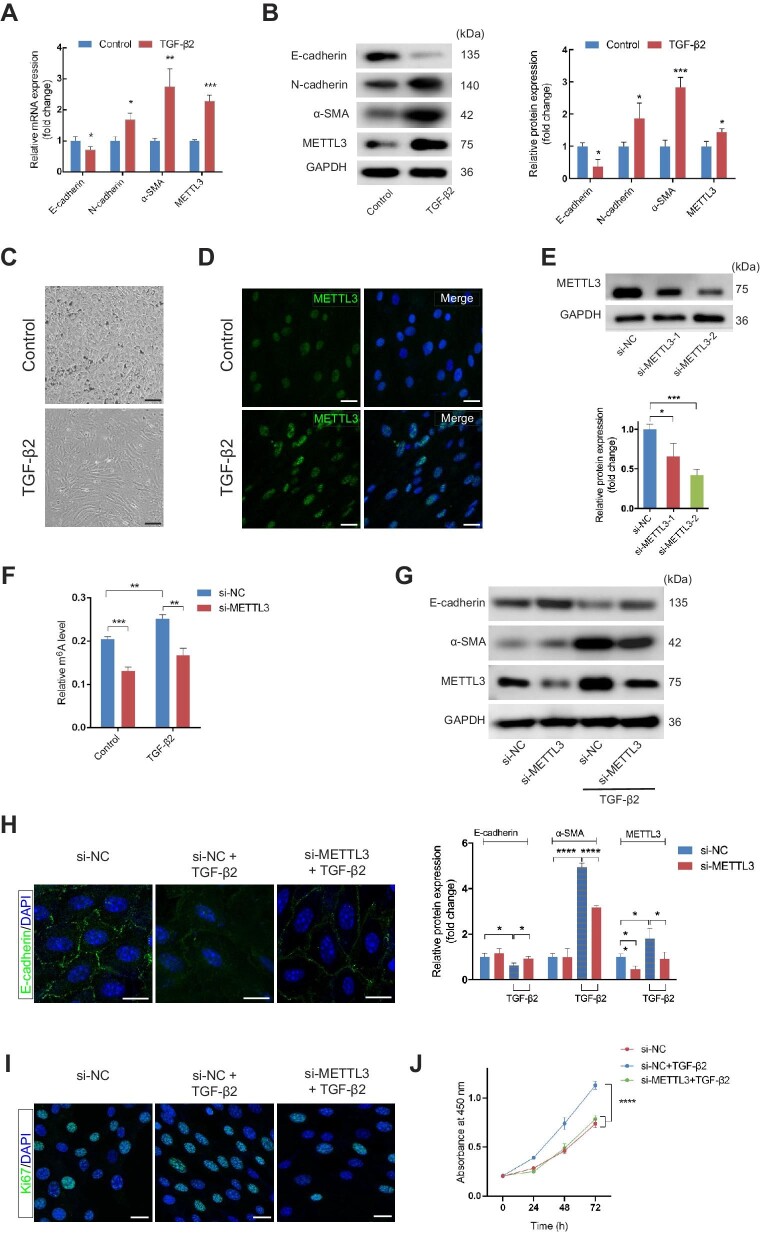
METTL3 is involved in primary mouse RPE cells undergoing EMT. (**A** and **B**) The mRNA (**A**) and protein (**B**) levels of EMT-related markers (E-cadherin, N-cadherin, and α-SMA) and METTL3 in primary mouse RPE cells with and without 10 ng/ml TGF-β2 for 48 h. (**C**) Phase-contrast microscopy images of RPE cells showed the spindle fibroblast-like morphology induced by TGF-β2 application. Scale bar, 50 µm. (**D**) Immunofluorescence analysis demonstrated increased METTL3 in RPE cells treated with TGF-β2. Scale bar, 25 µm. (**E**) Primary RPE cells were transfected with si-NC, si-METTL3-1, or si-METTL3-2. METTL3 protein expression was quantified by western blotting after 48 h. (**F**) Primary RPE cells were transfected with si-NC or si-METTL3-2 for 48 h before the application of TGF-β2 or not. Then, total RNA was extracted to assess the global m^6^A level. (**G**) Western blotting analysis of E-cadherin and α-SMA was performed to determine the effect of METTL3 inhibition on TGF-β2-induced EMT in primary RPE cells. (**H**) Immunofluorescence analysis of E-cadherin in RPE cells confirmed that the loss of E-cadherin caused by TGF-β2-induced EMT was partially restored by METTL3 deficiency. Scale bar, 25 µm. (**I**) Ki67-positive cells were reduced after METTL3 inhibition. Scale bar, 25 µm. (**J**) CCK8 assay showed that cell proliferation was inhibited by METTL3 knockdown. Data present mean ± SD of three independent experiments. Student's *t*-test for two independent groups and two-way ANOVA tests for CCK8 assay, *P < 0.05, **P < 0.01, ***P < 0.001, ****P < 0.0001.

EMT of RPE cells was observed in CNV tissues from patients and mouse models of subretinal fibrosis (Hirasawa et al., 2011; Kimura et al., 2015; Ishikawa et al., 2016b; Kobayashi et al., 2019; Liu et al., 2019; Wu et al., 2019a; Little et al., 2020). TGF-β is considered to be a potent EMT inducer for RPE cells and is significantly upregulated in RPE cells in the macula of nAMD patients (Amin et al., 1994; Kliffen et al., 1997). Recent evidence also indicated increased expression of TGF-β2 in subretinal fibrosis in the laser-induced CNV models (Little et al., 2020). Therefore, we treated primary mouse RPE cells with 10 ng/ml TGF-β2 for 48 h to induce EMT. RPE cells stimulated with TGF-β2 showed decreased expression of E-cadherin and increased mesenchymal markers at both mRNA and protein levels (Figure 3A and B). RPE cells showed spindle fibroblast-like morphology under phase-contrast microscopy after TGF-β2 application (Figure 3C). Meanwhile, the mRNA and protein levels of METTL3 were upregulated, along with total m^6^A levels (Figure 3A and B; Supplementary Figure S3A). Elevated fluorescence intensity of METTL3 was also observed using confocal microscopy (Figure 3D). No other m^6^A-associated protein except METTL3 underwent changes in mRNA levels (Supplementary Figure S3B). YTHDF1 protein, which showed a slight increase on Day 14 of laser-induced CNV, remained unchanged in RPE cells treated by TGF-β2 (Supplementary Figure S3C).

To assess whether METTL3 was necessary during the EMT process, we transfected RPE cells with small interfering RNAs (siRNAs) to knock down METTL3. The expression of METTL3 was detected by western blotting. Compared with transfection of negative control siRNA (si-NC), transfection with si-METTL3-2 decreased METTL3 by more than half (Figure 3E). Therefore, si-METTL3-2 was selected for the subsequent studies. Knockdown of METTL3 downregulated m^6^A levels in mRNAs of RPE cells with or without TGF-β2 application (Figure 3F). TGF-β2-induced changes of EMT-related proteins were partially rescued in RPE cells transfected with si-METTL3 (Figure 3G). Consistently, knockdown of METTL3 reversed the change of immunofluorescent expression of E-cadherin induced by TGF-β2 (Figure 3H), suggesting that METTL3 was involved in primary mouse RPE cells undergoing EMT. Additionally, overexpression of METTL3 was applied to further validate its role in EMT of RPE cells. The results showed that overexpression of METTL3 enhanced EMT with or without TGF-β2 treatment (Supplementary Figure S4). The effect of METTL3 knockdown on proliferating capacity of primary RPE cells was determined by the cell counting kit-8 (CCK8) assay and Ki67 staining. The results suggested that the cell proliferation of RPE cells treated with TGF-β2 was downregulated after inhibiting the expression of METTL3 (Figure 3I and J).

### Widespread m^6^A modification of primary mouse RPE cells undergoing EMT

We performed m^6^A epitranscriptomic microarray to characterize the variations of m^6^A modification in RPE cells undergoing EMT. A combined analysis of gene expression and m^6^A modification is shown in Supplementary Figure S5A and B. The upper right quadrant in each of the two figures indicated an upregulated mRNA level with increased m^6^A methylation level and m^6^A quantity, respectively. Compared with the untreated RPE cells, a total of 85 genes showed increased m^6^A methylation level and 6670 genes showed increased mRNA level (fold change >2). We identified overlapping genes between the upregulated mRNA level and elevated m^6^A level and listed the top five genes, among which HMGA2 has been suggested as an important TF controlling EMT (Supplementary Figure S5C).

### METTL3-mediated m^6^A modification enhances the stability of HMGA2 mRNA

**Figure 4:**
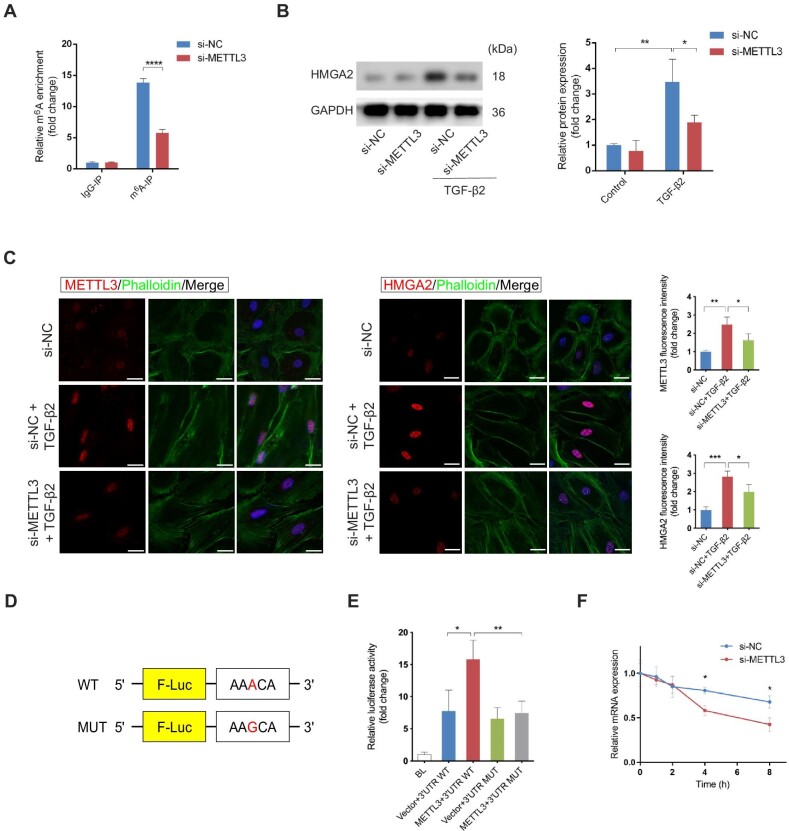
METTL3-mediated m^6^A modification enhances the stability of HMGA2 mRNA. (**A**) MeRIP–qRT-PCR with anti-m^6^A antibody was performed to determine the m^6^A enrichment of HMGA2 mRNA with or without METTL3 knockdown in primary RPE cells. (**B**) Western blotting analysis showed that HMGA2 protein level was induced by TGF-β2 and inhibited upon METTL3 knockdown. (**C**) Immunofluorescence analysis confirmed the reduced expression of HMGA2 in METTL3-knockdown RPE cells (*n* = 15 for each group). Actin cytoskeleton was visualized by phalloidin staining, which targets F-actin. Scale bar, 25 µm. (**D**) Schematic representation of pmirGLO dual-luciferase vectors fused with WT or MUT HMGA2 3′UTR. (**E**) 3′UTR WT or 3′UTR MUT reporters were transfected into RPE cells along with empty vectors or METTL3 expression plasmid. Relative luciferase activity was measured after 48 h. (**F**) After transfection with either si-METTL3 or si-NC for 48 h, primary RPE cells were treated with Act-D to block transcription. HMGA2 mRNA was analyzed at the indicated times. Data present mean ± SD of three independent experiments. Student's *t*-test for two independent groups, one-way ANOVA tests for luciferase reporter assay and repeated measures, two-way ANOVA tests for HMGA2 mRNA decay experiment, *P < 0.05, **P < 0.01, ***P < 0.001, ****P < 0.0001.

We screened the genes listed in the m^6^A epitranscriptomic microarray data and found the gene HMGA2 showing a 2.84-fold m^6^A change and a 68.07-fold mRNA change compared to the control group, respectively. The gene HMGA2 codes a non-histone architectural TF that positively regulates the EMT process in a series of fibrotic diseases (type 2 EMT) and cancer metastasis (type 3 EMT), which might be the main target of METTL3 in EMT of RPE cells (Liang et al., 2014; Hou et al., 2018; Zhu et al., 2019; Song et al., 2021; Wang et al., 2021). Results of the m^6^A epitranscriptomic microarray showed the sequence of HMGA2 mRNA modified by m^6^A was located in 3′UTR (CAAUACCUUCUCUAGUGGAUUAUCACUGUCUGCACAAUAAACAUAACAGCCUCUGUGGGC). Given that m^6^A modification selectively occurs within the consensus motif RRACH (R = G or A; H = A, C, or U), we hypothesized that AAACA within the above sequence was methylated by METTL3. Therefore, we applied methylated RNA immunoprecipitation followed by qRT-PCR (MeRIP–qRT-PCR) to validate the enriched m^6^A modification of HMGA2 mRNA. We noted a 2.8-fold increase in the m^6^A level of HMGA2 mRNA following METTL3 overexpression (Figure 4A). To verify the effect of METTL3 on the target gene HMGA2, we detected the protein level of HMGA2 in METTL3-knockdown RPE cells with or without TGF-β2. As represented in Figure 4B, the elevation of HMGA2 induced by TGF-β2 was diminished by METTL3 knockdown. Immunofluorescence analysis verified the reduced expression of HMGA2 in METTL3-knockdown RPE cells (Figure 4C). Besides, the untreated RPE cells displayed a cobblestone-like appearance with F-actin visualized by phalloidin staining, whereas TGF-β2-induced cells exhibited the spindle-shaped morphology and remodeled actin cytoskeleton (Figure 4C).

To determine whether m^6^A modification on HMGA2 3′UTR is essential for METTL3-regulated HMGA2 expression, we constructed luciferase reporters containing WT or mutant (MUT) HMGA2 3′UTR (Figure 4D). The luciferase activity of the WT group was intensified by overexpressing METTL3, while that of the MUT group remained unchanged (Figure 4E). Next, we investigated the mechanisms by which m^6^A methylation regulated HMGA2 expression. Considering that both mRNA and protein levels of HMGA2 increased during TGF-β2-induced EMT, we explored whether the m^6^A modification affected the stability of HMGA2 mRNA. We found that knockdown of METTL3 accelerated the degradation of HMGA2 mRNA (Figure 4F), and a consistent result was also observed at the protein level (Supplementary Figure S6). Collectively, METTL3 upregulated the expression of HMGA2 through stabilizing HMGA2 mRNA via m^6^A modifications in 3′UTR.

### HMGA2 promotes EMT via induction of SNAIL

**Figure 5:**
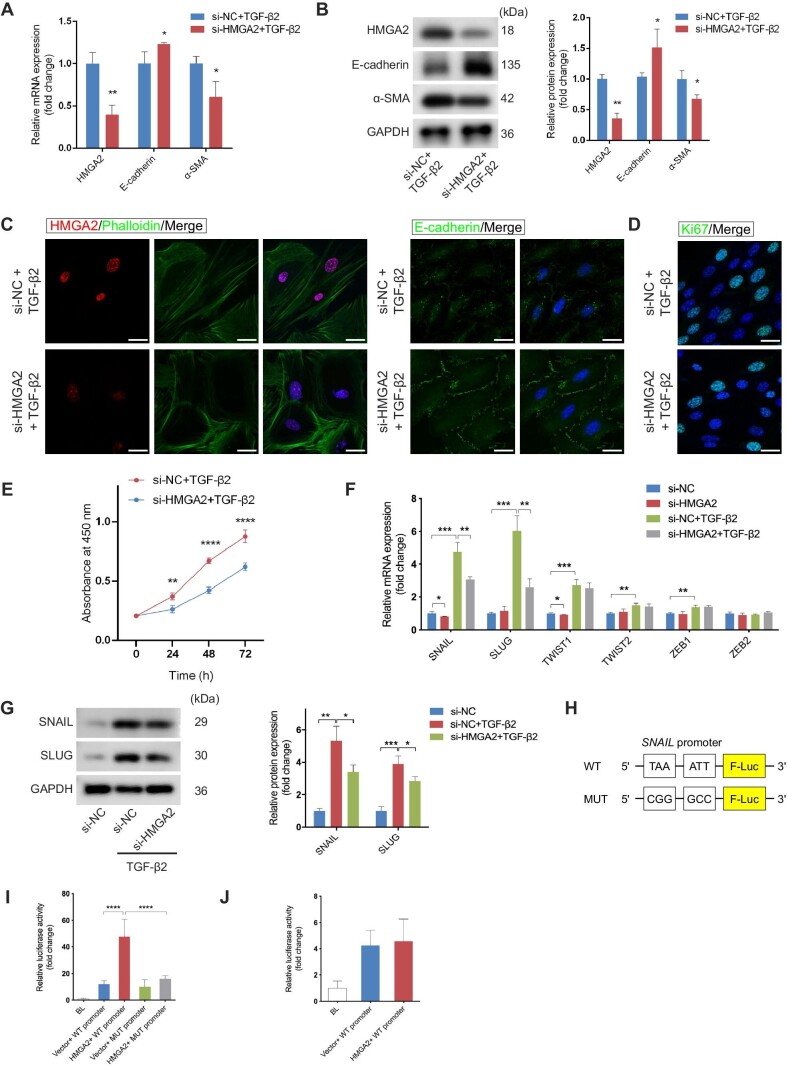
HMGA2 promotes EMT via induction of SNAIL. Primary RPE cells were transfected with si-NC or si-HMGA2 for 48 h following TGF-β2 treatment. (**A** and **B**) The mRNA (**A**) and protein (**B**) levels of HMGA2, E-cadherin, and α-SMA were quantified to detect the effect of HMGA2 inhibition on TGF-β2-induced EMT. (**C**) Immunofluorescence analysis confirmed the expression of E-cadherin and HMGA2 in the presence of si-HMGA2. Scale bar, 25 µm. (**D**) Ki67-positive cells were reduced after HMGA2 inhibition. Scale bar, 25 µm. (**E**) CCK-8 assay showed that cell proliferation was inhibited by HMGA2 knockdown. (**F**) The mRNA levels of several EMT-TFs were examined in the control and HMGA2-knockdown cells with or without TGF-β2 stimulation. (**G**) The expression of SNAIL and SLUG was monitored at protein levels. (**H**) Diagrammatic sketch of WT and MUT *SNAIL* promoters. (**I**) Luciferase reporter assays were carried out in mouse RPE cells, which were co-transfected with the WT or MUT *SNAIL* promoters and empty vectors or HMGA2 expression plasmid. (**J**) Luciferase reporter assays were performed to determine whether the *SLUG* promoter activity would change as a result of overexpressing HMGA2 in mouse RPE cells. Data present mean ± SD of three independent experiments. Student's *t*-test for two independent groups, one-way ANOVA tests for luciferase reporter assay, and two-way ANOVA tests for CCK8 assay, *P < 0.05, **P < 0.01, ***P < 0.001, ****P < 0.0001.

Although HMGA2 is known as a critical transcriptional factor regulating EMT in cancers, its role in RPE cells undergoing EMT has not been elucidated thus far (Liang et al., 2014; Zhu et al., 2019; Song et al., 2021; Wang et al., 2021). As shown in Figure 5A and B, silencing of HMGA2 suppressed TGF-β2-induced EMT at both mRNA and protein levels. It was also validated by immunofluorescence staining that HMGA2 contributed to the loss of E-cadherin (Figure 5C). Besides, inhibition of HMGA2 downregulated proliferation of cells treated with TGF-β2 (Figure 5D and E). Conversely, overexpression of HMGA2 promoted EMT of RPE cells after METTL3 inhibition (Supplementary Figure S7A). Furthermore, WT or mutant HMGA2 was expressed in HMGA2 pre-knocked cells to directly evaluate the effect of the mutation in m^6^A sites of HMGA2 in the process of EMT. As expected, the mutation led to a reduced protein level of HMGA2 and attenuated EMT of RPE cells, supporting the critical role of the METTL3–m^6^A–HMGA2 axis in EMT of RPE (Supplementary Figure S7B).

Next, we aimed to clarify the molecular mechanisms by which HMGA2 regulated EMT. Previous studies reported that HMGA2 activates several EMT-TFs by binding to their AT-rich DNA sequences, thereby affecting the expression of EMT-related proteins (Thuault et al., 2008; Tan et al., 2012; Li et al., 2014b, 2017). We examined the mRNA expression of EMT-TFs by qRT-PCR. The results showed that HMGA2 knockdown could partially counteract the increase of SNAIL and SLUG induced by TGF-β2 (Figure 5F). Although TWIST1 was reduced by silencing of HMGA2 in untreated RPE cells, its increase induced by TGF-β2 could not be rescued (Figure 5F). The protein level of SNAIL and SLUG was further evaluated, and the result showed that both of them were drastically upregulated after TGF-β application, which was attenuated in the context of deletion of HMGA2 (Figure 5G). Next, we investigated whether HMGA2 directly transactivated SNAIL and SLUG. According to Thuault et al. (2008), two AT-rich sequences in the *SNAIL* promoter from −131 to −92 were identified as HMGA2-binding sites. Therefore, we constructed a reporter plasmid using the *SNAIL* promoter containing the mutation of these two sequences (Figure 5H). We transfected primary mouse RPE cells with reporter plasmids containing WT or MUT *SNAIL* promoter and HMGA2 expression plasmid. The luciferase reporter assays showed that the mutations led to reduced SNAIL transcription activity in the context of overexpression of HMGA2, demonstrating that SNAIL was transcriptionally activated by HMGA2 (Figure 5I). To determine the combination between HMGA2 and the *SLUG* promoter, an HMGA2 expression plasmid and a reporter construct plasmid carrying a 3000-bp DNA fragment upstream of the *SLUG* transcription start site were transfected into mouse primary RPE cells. The results showed that SLUG transcription activity was not upregulated after HMGA2 was overexpressed, indicating that SLUG might not be directly activated by HMGA2 (Figure 5J).

### METTL3 silencing inhibits EMT and subretinal fibrosis

**Figure 6:**
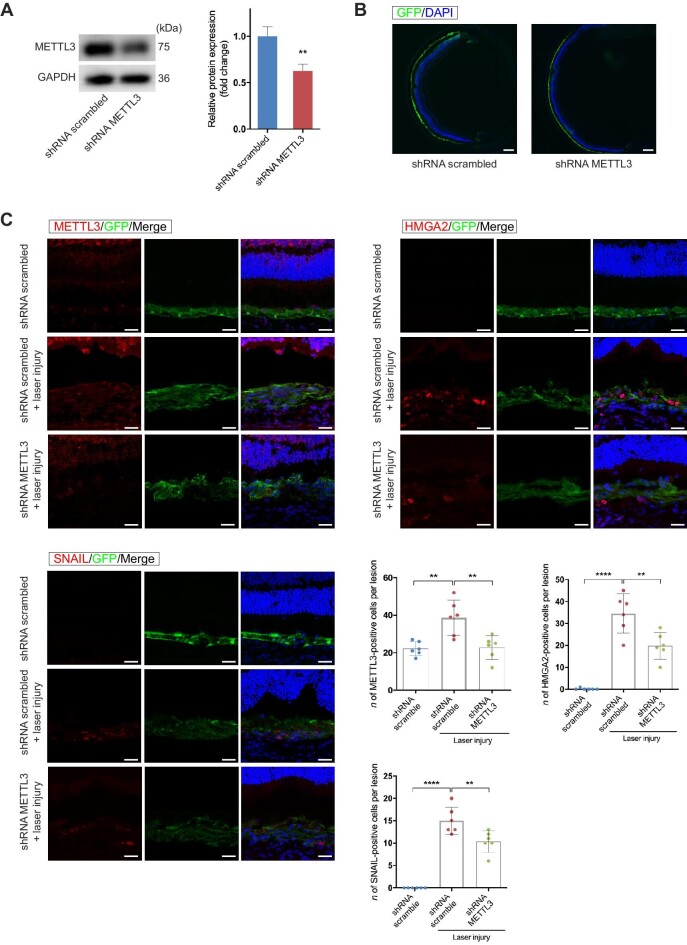
The expression levels of METTL3, HMGA2, and SNAIL are reduced following METTL3 knockdown in RPE cells through subretinal injection of AAV2/2.shRNA-METTL3. (**A**) Three weeks after subretinal injection of AAV vectors, RPE–choroid complexes were collected to measure the METTL3 protein level. Scale bar, 200 µm. (**B**) GFP staining showed that AAV vectors were effectively transduced into RPE cells. (**C**) Laser photocoagulation was applied 3 weeks after AAV transduction. Immunofluorescence assays were performed to evaluate the expression of METTL3, HMGA2, and SNAIL in subretinal fibrosis with or without METTL3 silencing on Day 28 after laser photocoagulation (*n* = 6 for each group). Scale bar, 50 µm. Data present mean ± SD of three independent experiments. Student's *t*-test, **P < 0.01, ****P < 0.0001.

To examine the effect of silencing METTL3 on CNV-associated subretinal fibrosis, we constructed adeno-associated virus 2/2 (AAV2/2) vectors carrying green fluorescent protein (GFP) to deliver shRNA targeting METTL3 in RPE cells in 3-week-old C57BL/6 mice. AAV2/2 vector containing an shRNA sequence specific to METTL3 (AAV2/2.shRNA-METTL3) and control vector (AAV2/2.shRNA scrambled) were delivered into RPE cells by subretinal injection, respectively (Supplementary Figure S8). After 21 days, the knockdown efficiency of METTL3 was evaluated by western blotting (Figure 6A). The transduction efficiency was observed by immunofluorescent staining. The results of GFP staining showed that AAV vectors were effectively transduced into RPE cells (Figure 6B). Subsequently, we performed immunofluorescence assays to evaluate the expression of HMGA2 and SNAIL in laser-induced subretinal fibrosis with or without METTL3 silencing (Figure 6C). The numbers of HMGA2-expressing and SNAIL-expressing cells were obviously reduced within the GFP-positive area, indicating the inhibition of HMGA2 and SNAIL expression by METTL3 knockdown.

**Figure 7:**
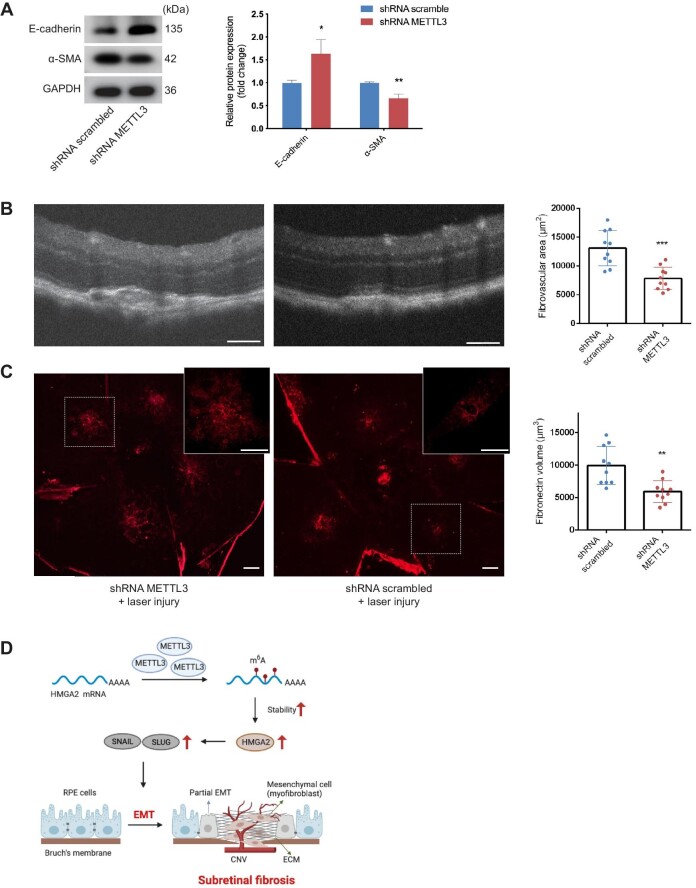
METTL3 silencing inhibits EMT and subretinal fibrosis induced by laser photocoagulation. (**A**) Western blotting analysis of E-cadherin and α-SMA was conducted to evaluate the EMT process upon METTL3 knockdown *in vivo*. (**B**) OCT images of individual lesions were acquired 28 days after laser induction, presenting the smaller area of subretinal lesions caused by METTL3 knockdown (*n* = 10 for each group). Scale bar, 100 µm. (**C**) Immunofluorescence staining on RPE–choroid flatmounts showed a reduction in the volume of Fibronectin-positive lesions in the shRNA METTL3 group (*n* = 10 for each group). Scale bar, 200 µm. (**D**) Schematic representation of the METTL3–m^6^A–HMGA2 axis in subretinal fibrosis. During the formation of subretinal fibrosis, METTL3 is upregulated in RPE cells undergoing EMT. METTL3 promotes the stability of HMGA2 mRNA via m^6^A modification in 3′UTR, and the METTL3–m^6^A–HMGA2 axis positively regulates the induction of potent EMT-TFs, including SNAIL and SLUG. Data present mean ± SD. Student's *t*-test, *P < 0.05, **P < 0.01, ***P < 0.001.

Next, we investigated whether METTL3 deficiency inhibited EMT and subretinal fibrosis. On Day 28 after laser induction, proteins were extracted from RPE–choroid complexes to detect EMT-related markers. Results showed that knockdown of METTL3 significantly suppressed the EMT process (Figure 7A). We analyzed the size of subretinal fibrosis in different ways. Optical coherence tomography (OCT) images were captured to measure the subretinal fibrovascular area at the maximum point. The area of the subretinal lesion was significantly smaller in the AAV2/2.shRNA-METTL3 group compared with that in the AAV2/2.shRNA scrambled group (Figure 7B). Furthermore, RPE–choroid flatmounts were stained with anti-Fibronectin antibody, another major component of ECM. The volume of the Fibronectin-positive lesion was reduced in the AAV2/2.shRNA-METTL3 group (Figure 7C). Taken together, these results indicated that METTL3 deficiency in RPE cells downregulated the expression of HMGA2 and SNAIL induced by laser injury, which inhibited EMT and subretinal fibrosis induced by laser photocoagulation (Figure 7D).

## Discussion

Subretinal fibrosis is the end-stage sequela of nAMD, predicting a poor visual prognosis. There is an urgent need for effective strategies to prevent fibrosis. In the present study, we established a laser-induced CNV mouse model, in which we observed the formation of EMT-mediated subretinal fibrosis and the elevation of METTL3 within RPE cells. The involvement of METTL3 in EMT of RPE cells was confirmed *in vitro*. After inhibiting METTL3 in primary mouse RPE cells, TGF-β2-induced EMT and proliferation were significantly suppressed. m^6^A epitranscriptomic microarray was conducted to map the change of m^6^A modification in TGF-β2-treated RPE cells. We found that METTL3-mediated m^6^A modification promoted HMGA2 expression by enhancing mRNA stability and then regulated the potent EMT-TF SNAIL. We provide compelling evidence demonstrating that the METTL3–m^6^A–HMGA2 axis is essential for the formation of subretinal fibrosis and EMT of RPE cells.

Subretinal fibrosis secondary to nAMD is an uncontrolled result of tissue repair and wound healing. A variety of pathological processes have been implicated, including the recruitment and activation of fibroblasts and inflammatory cells, leading to the excessive deposition of ECM. However, the origin of myofibroblasts and the underlying mechanisms of ECM deposition remain elusive. A study published in 1996 reported that in surgically excised fibrovascular membranes, many cells with double staining of cytokeratin and α-SMA were detected, representing the transdifferentiated RPE cells (Lopez et al., 1996). Additionally, the transdifferentiated RPE formed a gradient distribution, probably reflecting the transition process (Lopez et al., 1996). Up to date, more and more studies demonstrated that RPE cells were one of the main sources of myofibroblasts through the EMT process and contributed to subretinal fibrosis formation, based on findings derived from clinical samples and preclinical research (Hirasawa et al., 2011; Little et al., 2020). Transdifferentiated RPE cells were observed in CNV tissues from patients and mouse models of subretinal fibrosis (Hirasawa et al., 2011; Kimura et al., 2015; Ishikawa et al., 2016b; Kobayashi et al., 2019; Liu et al., 2019; Wu et al., 2019a; Little et al., 2020). Ishikawa et al. (2016b) reported that αB-crystallin facilitated EMT of RPE cells and development of fibrosis through modulation of SMAD4 and SMAD5 nuclear translocation. Wu et al. (2019a) showed that Galectin-1, a galactoside-binding lectin family protein, induced the phosphorylation of SMAD2 and promoted EMT-mediated subretinal fibrosis. Several therapeutic agents were proven to be effective at preventing subretinal fibrosis via regulating EMT of RPE cells (Kobayashi et al., 2019; Liu et al., 2019; Wu et al., 2019a). In our study, we observed the downregulation of the epithelial marker and upregulation of mesenchymal markers in a mouse model of subretinal fibrosis, as well as the partial co-localization of RPE65 and α-SMA, which strongly proved the presence of EMT during fibrosis formation. Moreover, we identified the m^6^A ‘writer’ METTL3 as a critical epigenetic regulator in EMT-mediated subretinal fibrosis.

Besides RPE cells, multiple cell types have been reported to directly transdifferentiate to myofibroblasts and contribute to the development of subretinal fibrosis, such as glial cells, macrophages, pericytes, and endothelial cells (Little et al., 2018, 2020; Luo et al., 2018). Nevertheless, EMT could be considered to play a key role, given that our and other studies have shown that targeting the EMT process effectively inhibited subretinal fibrosis (Kobayashi et al., 2019; Liu et al., 2019; Wu et al., 2019a).

The mechanism of EMT of RPE cells has been widely studied. However, the epigenetic regulation in EMT of RPE cells remains largely unknown. RNA m^6^A modification has attracted increasing attention as the most abundant internal modification in eukaryotes. Several studies reported that METTL3-mediated m^6^A modification induced the activation of myofibroblasts and promoted fibrosis formation in fibrotic diseases of various organs, such as the heart, liver, lung, and kidney (Han et al., 2020; Zhu et al., 2020; Li et al., 2020a; Liu et al., 2020a). A recent study showed that METTL3 was decreased in human PVR membranes and TGF-β2-treated ARPE-19 cells via wnt/β-catenin pathway (Ma et al., 2021). In our study, we demonstrated METTL3 as a positive regulator of EMT-mediated subretinal fibrosis. The contrary conclusion about the effect of METTL3 on EMT of RPE cells may be due to the different *in vivo* and *in vitro* models used in our study, implying different mechanisms of EMT between PVR and nAMD, or between ARPE-19 cell lines and primary mouse RPE cells. In this study, we found that upregulated METTL3 was located in RPE65-positive and α-SMA-positive cells, which are transdifferentiated RPE cells. Besides, METTL3 was upregulated on Days 14 and 28 after laser injury but remained unchanged on Day 7, implying that METTL3 might not be involved in CNV formation. More notably, loss of METTL3 diminished the expression of the EMT-related marker and attenuated subretinal fibrosis. We herein first revealed the epigenetic regulation of METTL3-mediated m^6^A modification on EMT-mediated subretinal fibrosis.

Mechanistically, we performed m^6^A epitranscriptomic microarray and verified the results through *in vitro* experiments, demonstrating that METTL3 epigenetically enhanced HMGA2 expression via the modulation of HMGA2 mRNA stability. Expression of HMGA2 is mainly restricted to the embryonic stage and is undetectable or at low levels in normal adult tissues (Rogalla et al., 1996). The overexpressed HMGA2 in adult somatic cells correlates with tumorigenesis and metastasis (Zhang et al., 2019; Mansoori et al., 2020; Li et al., 2020b). Moreover, HMGA2 has been increasingly reported to participate in fibrotic diseases as a key positive regulatory factor in EMT (Liang et al., 2014; Hou et al., 2018; Zhu et al., 2019; Song et al., 2021). Recently, it has been reported that HMGA2 was upregulated in RPE cells undergoing EMT induced by TGF-β2 (Sripathi et al., 2019). However, the role of HMGA2 in the regulatory network and the underlying mechanism remain to be clarified. Our study not only confirmed the upregulated HMGA2 during EMT in RPE cells, which was consistent with previous research results, but also identified HMGA2 as the main target of METTL3 in RPE cells undergoing EMT through *in vitro* and *in vivo* experiments. Recent studies demonstrated that METTL3 indirectly promotes HMGA2 expression by regulating the m^6^A modification of circHPS5 and MALAT1 in tumor cells (Rong et al., 2021; Zhao et al., 2021). Considering that m^6^A modifications are widely present in RNAs, we cannot exclude the possibility that METTL3 promotes HMGA2 via the regulation of lncRNAs or circRNAs in EMT of RPE. However, we transfected mutant HMGA2 expression plasmids into HMGA2 pre-knocked cells and then found a reduced protein level of HMGA2 and attenuated EMT of RPE cells. Therefore, METTL3-mediated m^6^A modification of HMGA2 mRNA plays a key role in EMT of RPE.

The m^6^A reader proteins are vital as they bind to m^6^A-modified RNAs and regulate their metabolism, including RNA stability. In our study, although YTHDF1 protein showed a slight increase on Day 14 of laser-induced CNV, it remained unchanged in RPE cells treated by TGF-β2. With several different m^6^A readers (YTHDF1/2/3, IGF2BP1/2/3, eIF3, HNRNPA2B1, Prrc2a, etc.) being identified, further investigation is necessary to figure out the specific m^6^A readers of HMGA2 mRNA (Tong et al., 2018; Wu et al., 2019b).

Previous studies reported that HMGA2 activated EMT-TFs through direct binding to the promoter, such as SNAIL, SLUG, and TWIST (Thuault et al., 2006; Zhang et al., 2019). For instance, Thuault et al. (2008) identified HMGA2 as the transcriptional activator of SNAIL during EMT of mouse mammary epithelial NMuMG cells and further revealed the bind sites in the proximal region of the *SNAIL* promoter. HMGA2 also directly induces the target gene SLUG to promote EMT, migration, and proliferation of colon cancer cells (Li et al., 2014b). During the progression of gastric cancer, HMGA2 transactivates TWIST1 to regulate EMT of gastric cancer cells (Li et al., 2017). As for EMT of RPE cells, increased expression of HMGA2 was reported, but the downstream mechanism has not been explored so far. In this study, we measured the expression of these TFs in HMGA2-knockdown RPE cells and found that SNAIL and SLUG, which belong to the SNAIL family of zinc-finger TFs and are well recognized as having key roles in EMT of RPE cells (Shu et al., 2020), were dramatically reduced at mRNA and protein levels after HMGA2 knockdown. Luciferase reporter assays were carried out to explore the regulatory mechanism of HMGA2 on SNAIL and SLUG. The results showed that HMGA2 transcriptionally activated SNAIL but not SLUG in primary mouse RPE cells, indicating that SLUG might be indirectly regulated by HMGA2, which needs further investigation. Taken together, we confirmed HMGA2 as an important transcriptional activator of SNAIL, a core regulatory factor in EMT of RPE cells.

In conclusion, we highlighted the critical role of METTL3-mediated m^6^A modification in EMT of RPE cells and subretinal fibrosis. METTL3 enhanced the expression of HMGA2 in an m^6^A-dependent manner, which subsequently transactivated the EMT-TF SNAIL. We provided a novel epitranscriptomic mechanism underlying EMT of RPE cells, suggesting the METTL3–m^6^A–HMGA2 axis as a promising therapeutic target for the treatment of subretinal fibrosis secondary to nAMD.

## Materials and methods

### Laser-induced CNV model

C57BL/6 mice were provided by the Laboratory Animal Center, Shanghai General Hospital. All animal experiments were performed under the approval of the Animal Research Committee at Shanghai Jiaotong University and following the Association for Research in Vision and Ophthalmology Statement. Eight-week-old female mice were used to establish laser-induced CNV models as previously described (Luo et al., 2018). Briefly, mice were anesthetized with 1.5% sodium pentobarbital, and the pupils were dilated with 1% tropicamide. An argon laser (532 nm, 120 mW, 100 ms, and 50 µm; Visuals 532S; Carl Zeiss Meditec) with a slip lamp delivery system was used to make four laser spots around the optic nerve for each eye.

### Cell culture, treatment, and transfection

Primary mouse RPE cell isolation and culture were the same as in our previous study (Sun et al., 2020). After the anterior segment and retina were removed, the RPE sheets were peeled off and treated with 0.25% trypsin for 20 min (Gibco). Then, the single-cell suspension was collected and cultured in Dulbecco's modified Eagle's medium/Ham's F-12 medium (Gibco) containing 10% fetal bovine serum, 1% non-essential amino acids, and 1% HEPES (Gibco). The medium was changed every 3 days. The passage 0 cells were re-plated after confluence at a split of 1:1 for the control group. For the EMT group, cells were passaged at a 1:3 ratio and treated with recombinant mouse TGF-β2 (R&D Systems) at a concentration of 10 ng/ml to model the EMT of RPE *in vivo*. RPE cells were cultivated in serum-free medium for 12 h before TGF-β2 application. RPE cells were used at passage 1 in all experiments, because repeated passage caused EMT in primary RPE cells (Shih et al., 2017).

siRNAs were applied to reduce the expression of METTL3 and HMGA2. METTL3 expression plasmid and HMGA2 expression plasmid were used for METTL3 and HMGA2 overexpression, respectively. Cells were transfected with siRNAs or plasmids using TransIT-X2 Dynamic Delivery System (Mirus Bio) according to the manufacturer's protocol. The sequences of siRNAs are listed in Supplementary Table S1. All siRNAs were purchased from Shanghai GenePharma. The pLenti6.3-MCS-IRES-EGFP vectors overexpressing Lenti ORF clones of mouse METTL3 (NM_019721) and mouse HMGA2 (NM_001347170.1) were purchased from Shanghai Novobio.

### qRT-PCR

Total RNA was extracted from primary mouse RPE cells or RPE–choroid complexes with a Total RNA Extraction Kit (Tiangen) and then quantified using NanoDrop 2000c spectrophotometer (Thermo Fisher Scientific). After generating cDNA using RT Master Mix (TaKaRa), mRNA levels were quantified with qRT-PCR using SYBR Premix Ex Taq (TaKaRa) according to the manufacturer's protocol. The primer sequences are listed in Supplementary Table S2.

### Western blotting

The primary mouse RPE cells or RPE–choroid complex-affected retinas were lysed with radio-immunoprecipitation assay buffer (Beyotime) supplemented with protease inhibitors. Samples were separated with 10% SDS–PAGE gels and then transferred to polyvinylidene difluoride membranes (Merck Millipore). The membranes were probed with primary antibodies for E-cadherin (3195, Cell Signaling Technology), N-cadherin (13116, Cell Signaling Technology), α-SMA (ab5694, Abcam), Fibronectin (AB2033, Merck Millipore), GAPDH (10494–1-AP, Proteintech), METTL3 (ab195352, Abcam), HMGA2 (8179, Cell Signaling Technology), SNAIL (ab216347, Abcam), and SLUG (ab106077, Abcam). The membranes were incubated with secondary antibodies (Proteintech) for 1 h at room temperature and exposed with Amersham Imager 600 (General Electric Healthcare). ImageJ 1.53h (http://imagej.nihgov/ij) was used for densitometrical quantification.

### Immunofluorescence staining

Immunofluorescence assays were performed with retinal sections, RPE–choroid flatmounts, or primary RPE cells in 24-well slide chambers. Primary antibodies were used for staining against E-cadherin (3195, Cell Signaling Technology), N-cadherin (13116, Cell Signaling Technology), α-SMA (ab5694, Abcam), RPE65 (GTX13826, GeneTex), METTL3 (ab195352, Abcam), IB4 (DL-1207, Vector Laboratories), F4/80 (MCA497, Abd Serotec), Alexa Fluor^TM^ 488 Phalloidin (A12379, Thermo Fisher Scientific), Ki67 (ab15580, Abcam), HMGA2 (8179, Cell Signaling Technology), and GFP (ab290, Abcam).

To evaluate the volume of subretinal fibrosis, RPE–choroid flatmounts were stained with the antibody against Fibronectin (AB2033, Merck Millipore) on Day 28 after laser injury. The volume analysis was performed as previously described (Ye et al., 2015). Fibronectin was visualized using a confocal laser scanning microscope (TCS SP8; Leica Biosystems). Horizontal optical sections were taken at 1 µm intervals from the top to the bottom of the fibrosis lesion. The area of Fibronectin-positive lesion on each layer was measured using ImageJ software. The total area of each horizontal section was used as an index of Fibronectin volume. All laser spots in each eye were measured.

### Masson's trichrome staining

After fixation and embedding, eyes were cut into 5-µm-thick sections. The section with the largest area of visualized subretinal fibrosis was selected for Masson's trichrome staining. All laser spots in each eye were stained and measured. Collagen fibers were stained blue and quantified with ImageJ software. The results were shown as the percentage of the fibrotic area to the whole area of the field.

### RNA m^6^A quantification

Total RNA was extracted and quantified in the same way as in the ‘qRT-PCR’ section. The m^6^A modification level of total RNA was detected using the EpiQuik m^6^A RNA Methylation Quantification Kit (Epigentek). Briefly, 200 ng of the total RNA was added into assay wells and then reacted with capture antibody and detection antibody. The absorbance at 450 nm was measured in a microplate spectrophotometer, and the m^6^A levels were calculated based on the standard curve.

### CCK8 assay

CCK8 assay was performed to determine the effect of METTL3 or HMGA2 knockdown on the cell proliferation of TGF-β2-treated primary RPE cells. Primary RPE cells (passage 1, 5 × 10^3^ cells/well) were seeded into 96-plate wells. After siRNA transfection, TGF-β2 was applied to induce EMT. CCK8 solution (Beyotime) was added and incubated at 37°C for 2 h. The absorbance was measured at 450 nm on Days 1, 2, and 3.

### m^6^A epitranscriptomic microarray and microarray data analysis

Total RNAs from TGF-β2-treated and untreated groups (*n* = 3 for each group) were immunoprecipitated with m^6^A antibody. m^6^A-methylated RNAs were eluted from the immunoprecipitated magnetic beads as the ‘IP’ and labelled with Cy5, and the others were obtained from the supernatant as ‘Sup’ and labelled with Cy3 (Arraystar Super RNA Labeling Kit). The labelled RNAs were hybridized onto the Arraystar Mouse mRNA & lncRNA Epitranscriptomic Microarray (Arraystar). The arrays were scanned in two-color channels by an Agilent Scanner G2505C.

The captured array images were analyzed using Agilent Feature Extraction software (version 11.0.1.1). Raw intensities of IP and Sup were normalized with an average of log2-level spike-in RNA intensities. ‘m^6^A methylation level’ was calculated for the percentage of m^6^A methylation based on the IP- and Sup-normalized intensities. ‘m^6^A quantity’ was calculated for the m^6^A methylation amount based on the IP-normalized intensities. Differentially m^6^A-methylated RNAs between two comparison groups were identified by filtering with the fold change and statistical significance thresholds. Hierarchical clustering heatmap analysis was performed for differentially m^6^A-methylated RNAs.

### MeRIP–qRT-PCR

For MeRIP, 50 ng of the total RNA was kept as input, and the remaining 2 µg RNAs were bonded to m^6^A antibody (Synaptic Systems) and immunoprecipitated with Dynabeads® Protein A (Thermo Fisher Scientific). RNA quantification was conducted by qRT-PCR. The specific primer for MeRIP–qRT-PCR analysis according to the information from m^6^A epitranscriptomic microarray is shown in Supplementary Table S2.

### Luciferase report assay

According to the binding site provided by m^6^A epitranscriptomic microarray, the corresponding HMGA2 3′UTR (114730–113789), either WT or MUT (mutated A of the m^6^A sites with G), was cloned into the downstream of the pmirGLO dual-luciferase vector (Promega). Mouse *SNAIL* promoter (−625 to −1) including WT or MUT HMGA2-binding sites was inserted into the pGL6-TA-basic cloning site according to Thuault et al. (2008). Likewise, we generated a reporter plasmid carrying a 3000-bp DNA fragment upstream of the *SLUG* transcription start site.

Primary RPE cells were co-transfected with overexpression plasmid and WT or MUT reporter plasmid using the TransIT-X2 Dynamic Delivery System (Mirus). At 48 h after transfection, cells were collected to detect the activities of firefly luciferase and Renilla luciferase by a dual-luciferase reporter assay kit (Beyotime) according to the manufacturer's protocol.

### RNA decay experiment

After transfection with either si-METTL3 or si-NC for 48 h, primary RPE cells were treated with actinomycin D (Act-D, Santa Cruz) at 5 µg/ml for 2, 4, 6, or 8 h before collection and extraction for HMGA2 mRNA quantification by qRT-PCR.

### AAV2/2 construction and subretinal delivery

The more efficient one of two siRNAs targeting METTL3 was selected to construct AAV2/2.shRNA-METTL3. AAV2/2.shRNA-METTL3 and AAV2/2.shRNA scramble carrying a GFP reporter gene (titer 1.0 × 10^12^ GC/ml, both) were purchased from OBiO Technology.

To knock down METTL3 in RPE cells *in vivo*, recombinant AAV2/2 was delivered into the subretinal space of 3-week-old C57BL/6 mice. Animal anesthesia and pupil dilation were performed as described above. The eyes were covered with a drop of ofloxacin oculentum, acting as a magnifying glass. After creating a tunnel with a sharp 30-gauge needle at the corneal limbus, 1 µl of AAV2/2.shRNA-METTL3 or AAV2/2.shRNA scramble was injected into the subretinal space of the eyes, respectively, using a blunt 34-gauge needle. The detached retina indicated the successful injection. Laser photocoagulation for AAV-treated animals was performed 3 weeks after subretinal injection.

### OCT

OCT images were captured using a spectral-domain OCT system (Phoenix Micron) on Day 28 after laser irradiation. Animals were prepared as described above. The scan that passed through the center of the lesion with the largest area was selected for the measurement. Fibrovascular lesion areas were analyzed and quantified by using ImageJ software in a masked fashion.

### Statistical analysis

All data are presented as mean ± standard deviation (SD). GraphPad Prism (version 8; GraphPad Software) was used to create graphs and conduct statistical analysis. Comparisons between two independent groups were conducted using Student's *t*-test. One-way or two-way analysis of variance (ANOVA) with Tukey's multiple comparison post hoc test was applied to analyze the results of the three or more groups. *P* < 0.05 was considered statistically significant.

### Data availability statement

The m^6^A epitranscriptomic microarray data generated in this study are deposited on GEO under accession GSE178946.

## Supplementary Material

mjad005_Supplemental_File

## Notes

**Author contributions:** Y.W., T.L., and X.S. conceived and designed the study. Y.W., Y.C., J.L., X.L., J.C., J.S., Y.H., J.F., and P.H. performed the experiments and analyzed the data. Y.W. drafted the manuscript. M.J., T.Z., J.W., T.L., and X.S. critically revised the manuscript. All authors reviewed and approved the final manuscript.
