# Supplementary material for: METTL3-mediated m6A modification of HMGA2 mRNA promotes subretinal fibrosis and epithelial–mesenchymal transition
Source: J Mol Cell Biol. 2023 Mar 21;15(3):mjad005. doi: 10.1093/jmcb/mjad005 (PMC10603769; doi:10.1093/jmcb/mjad005)
Supplement: mjad005_Supplemental_File [file mjad005_supplemental_file.pdf]

Supplementary material

METTL3-mediated m6A modification of HMGA2 mRNA promotes subretinal fibrosis and epithelial–mesenchymal transition

Yuwei Wang[1,2,†], Yuhong Chen[1,2,†], Jian Liang[2,†], Mei Jiang[1,2], Ting Zhang[1,2], Xiaoling Wan[1,2], Jiahui Wu[1,2], Xiaomeng Li[1,2], Jieqiong Chen[1,2], Junran Sun[1,2], Yifan Hu[1,2], Peirong Huang[1,2], Jingyang Feng[1,2], Te Liu[5,*], and Xiaodong Sun[1,2,3,4,*]

[1] Department of Ophthalmology, Shanghai General Hospital, Shanghai Jiao Tong University School of Medicine, Shanghai 200080, China

[2] Shanghai Key Laboratory of Ocular Fundus Diseases, Shanghai 200080, China

[3] National Clinical Research Center for Eye Diseases, Shanghai 200080, China

[4] Shanghai Engineering Center for Visual Science and Photomedicine, Shanghai 200080, China

[5] Shanghai Geriatric Institute of Chinese Medicine, Shanghai University of Traditional Chinese Medicine, Shanghai 200031, China

[†] These authors contributed equally to this work.

* Correspondence to: Xiaodong Sun, Department of Ophthalmology, Shanghai General Hospital, Shanghai Jiao Tong University School of Medicine, 100 Haining Rd, Shanghai 200080, China; Tel: +86-21-63075768; E-mail: xdsun@sjtu.edu.cn; Te Liu, Shanghai Geriatric Institute of Chinese Medicine, Shanghai University of Traditional Chinese Medicine, 365 South Xiangyang Rd, Shanghai 200031, China; Tel: +86-13918909976; E-mail: liute1979@shutcm.edu.cn

Supplementary Figures

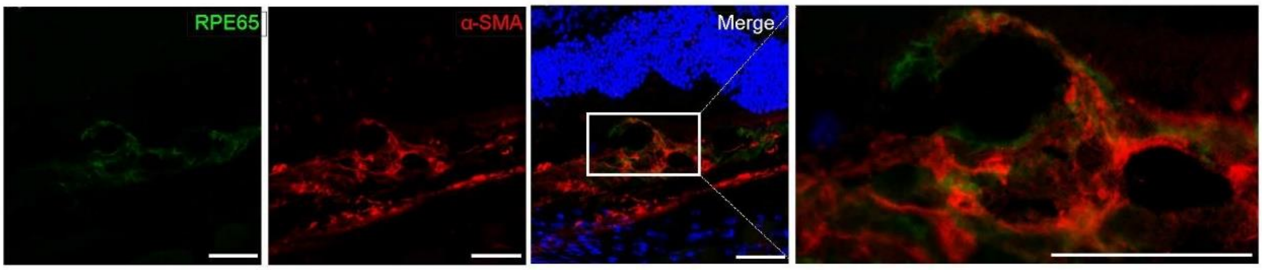

Supplementary Figure S1 RPE65$^+$α-SMA$^+$ double-positive cells were detected in the subretinal region. Scale bar, 25 μm.

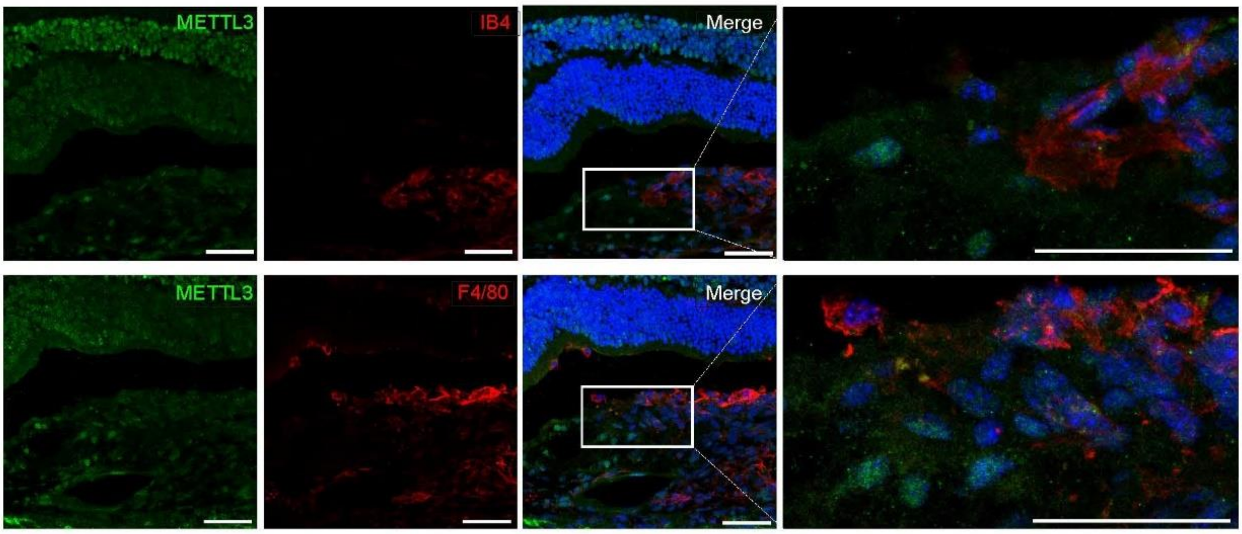

Supplementary Figure S2 Immunofluorescence co-staining showed that increased METTL3 was not located in the F4/80-positive or isolectin B4 (IB4)-positive cells in the subretinal region. Scale bar, 25 μm.

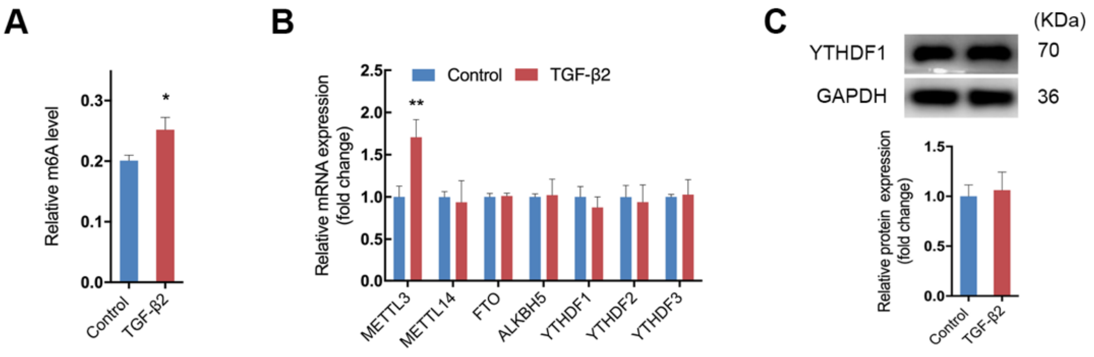

Supplementary Figure S3 Total m6A levels and expression of m6A RNA methylation regulators were measured in RPE cells undergoing EMT. (**A**) Total m6A levels of RPE cells with and without TGF-β2 treatment. (**B**) The mRNA levels of several m6A regulators were measured by qRT-PCR. (**C**) A western blot analysis of YTHDF1 in RPE cells. Data present mean ± SD of three independent experiments. Student's *t*-test for two independent groups, *$P < 0.05$, **$P < 0.01$.

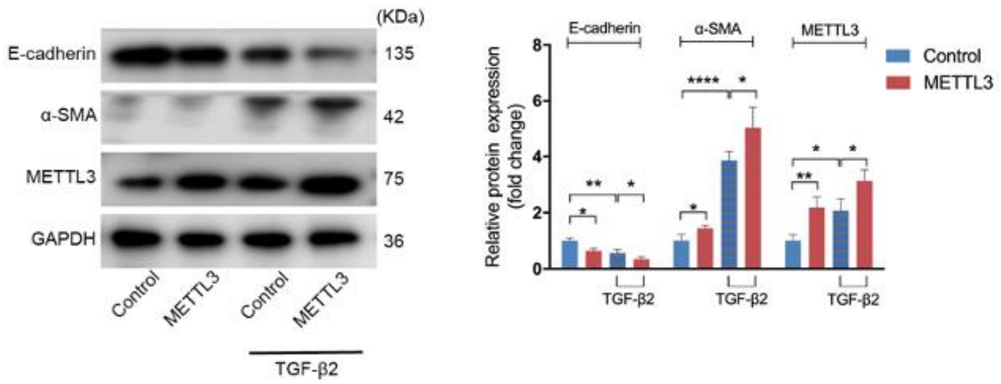

Supplementary Figure S4 Western blot analysis of E-cadherin and α-SMA was performed to determine the effect of METTL3 overexpression on the EMT of RPE cells. Data present mean ± SD of three independent experiments. Student's *t*-test for two independent groups, *$P < 0.05$, **$P < 0.01$, ****$P < 0.0001$.

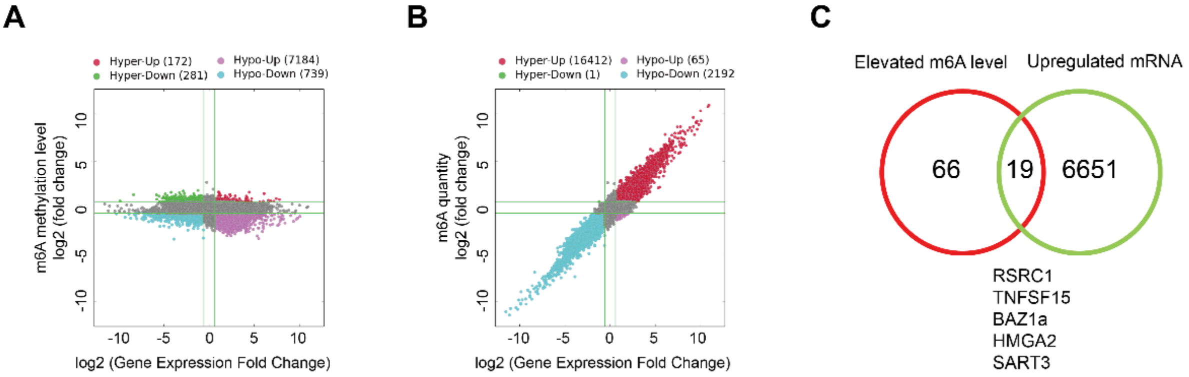

Supplementary Figure S5 Widespread m6A modification of primary mouse RPE cells undergoing EMT is revealed by m6A epitranscriptomic microarray. (**A**) A combined analysis of altered gene expression and m6A methylation level. (**B**) A combined analysis of altered gene expression and m6A quantity. (**C**) Overlapping of genes with elevated m6A level and mRNA level (fold change > 2).

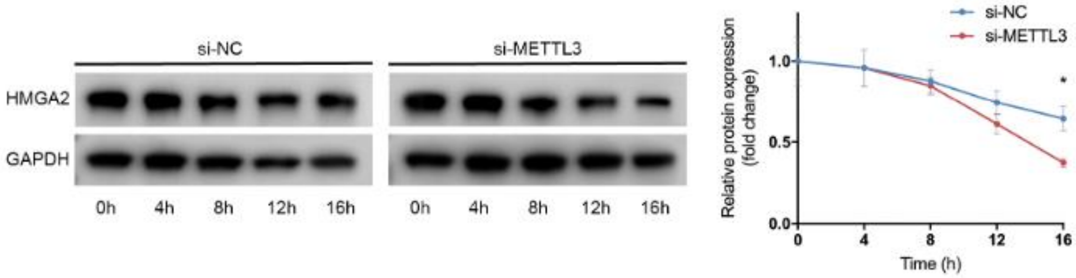

Supplementary Figure S6 Western blot analysis showed a faster decline in HMGA2 protein with METTL3 knockdown. Data present mean ±SD of three independent experiments. Repeated measures two-way ANOVA tests, *$P < 0.05$.

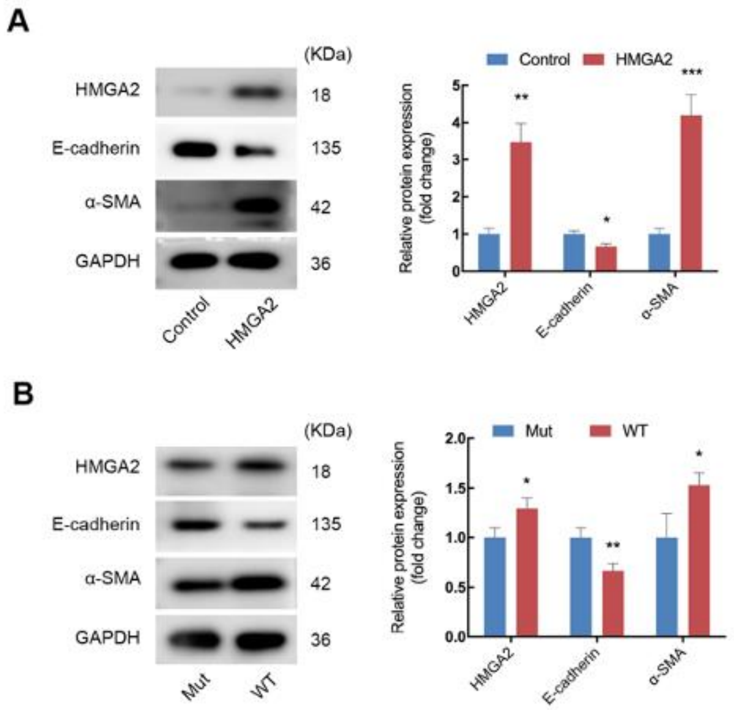

Supplementary Figure S7 The role of HMGA2 in the EMT of RPE cells. (**A**) After METTL3 inhibition, overexpression of HMGA2 promoted EMT of RPE cells. (**B**) WT or mutant HMGA2 expression plasmids were transfected into HMGA2 pre-knocked cells and the EMT was evaluated at the protein levels. Data present mean ± SD of three independent experiments. Student's *t*-test for two independent groups, **P* < 0.05, ***P* < 0.01, ****P* < 0.001.

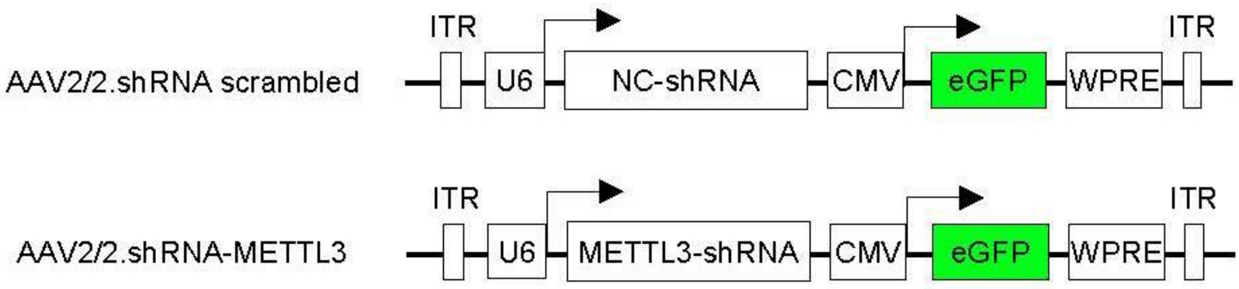

Supplementary Figure S8 Schematic representation of AAV2/2.shRNA scramble and AAV2/2.shRNA-METTL3 used to transfect RPE cells through subretinal injection.

Supplementary Tables

Supplementary Table S1 Sequences of siRNAs targeting METTL3 and HMGA2.

Gene	Sequence
METTL3	CUGCACUUCAGACGAAUUATT
METTL3 (better knockdown efficiency)	GCUACCGUAUGGGACAUUATT
HMGA2	GCCUUGAAGCAUCGGAGAUTT

Supplementary Table S2 Quantitative RT-PCR primers.

Gene	Forward primer	Reverse primer
CDH1	CAGGTCTCCTCATGGCTTTGC	CTTCCGAAAAGAAGGCTGTCC
CDH2	AGCGCAGTCTTACCGAAGG	TCGCTGCTTTCATACTGAACTTT
ACTA2	GTCCCAGACATCAGGGAGTAA	TCGGATACTTCAGCGTCAGGA
Fibronectin	ATGTGGACCCCTCCTGATAGT	GCCCAGTGATTTCAGCAAAGG
METTL3	GACTCTGGGCACTTGGAT	GTTGTGCTGGGCTTAGGG
METTL14	GAACCGTGAAGCGAAGCA	AGCCTGGCCTGATAGTGC
FTO	AGGATGAAAGTGAGGACGAG	TGGTGAAGAGGGATTGTTA
ALKBH5	TCAGCGACTCGGCACTTT	TTCATCAGCAGCATACCCAC
YTHDF1	CCCATCCCGTATCTCACT	CCTGTGCTGGTAAATGTTG
YTHDF2	GGTGGTGATGGTCTTCGCATAC	TTGGAGGTGCTGGAAAGTTCAG
YTHDF3	ATGAGAGAATGGGTCCTGGTT	GCCCAGAATCTCGTGACTCTTC
HMGA2	TGGGAGGAGCGAAATCTAAA	TCCCTGGAGAAGAGCTACG
SNAIL	CACACGCTGCCTTGTGTCT	GGTCAGCAAAAGCACGGTT
SLUG	TGGTCAAGAAACATTTCAACGCC	GGTGAGGATCTCTGGTTTTGGTA
TWIST1	GGACAAGCTGAGCAAGATTCA	CGGAGAAGGCGTAGCTGAG
TWIST2	CGCTACAGCAAGAAATCGAGC	GCTGAGCTTGTCAGAGGGG
ZEB1	GCTGGCAAGACAACGTGAAAG	GCCTCAGGATAAATGACGGC
ZEB2	ATTGCACATCAGACTTTGAGGAA	ATAATGGCCGTGTCGCTTCG
HMGA2 (for meRIP–qPCR)	TGTGCCCTCTGACTTCGTTC	CCCACAGAGGCTGTTA
